# Oxidation of Supported Nickel Nanoparticles: Effects of Lattice Strain and Vibrational Excitations of Active Sites

**DOI:** 10.3390/nano15181390

**Published:** 2025-09-10

**Authors:** Sergey Yu. Sarvadii, Andrey K. Gatin, Nadezhda V. Dokhlikova, Sergey A. Ozerin, Vasiliy A. Kharitonov, Dinara Tastaibek, Vladislav G. Slutskii, Maxim V. Grishin

**Affiliations:** 1 N.N. Semenov Federal Research Center for Chemical Physics, Russian Academy of Sciences (FRCCP RAS), Kosygina Str. 4, 119991 Moscow, Russia; akgatin@yandex.ru (A.K.G.); dohlikovanv@gmail.com (N.V.D.); sergeoz@yandex.ru (S.A.O.); vch.ost@mail.ru (V.A.K.); slutsky@list.ru (V.G.S.); mvgrishin68@yandex.ru (M.V.G.); 2 Institute of Cybernetics and Information Technology, Satbaev University (KazNRTU), Satbaeva Street 22A, Almaty 050013, Kazakhstan

**Keywords:** oxidation, nickel, HOPG, nanoparticles, STM spectroscopy, active site, surface migration, oxygen incorporation

## Abstract

This work investigated the oxidation in an atmosphere of N_2_O of different surface areas of single nickel nanoparticles deposited on highly oriented pyrolytic graphite (HOPG). Using scanning tunneling microscopy and spectroscopy, it was shown that oxide formation begins at the top of the nanoparticle, while the periphery is resistant to oxidation. The active site of oxygen incorporation is a vibrationally excited group of nickel atoms, and the gap between them is the place where an oxygen adatom penetrates. The characteristic time of vibrational relaxation of the active site is 10^−9^–10^−7^ s. The reason for the oxidation resistance is the deformation of the nanoparticle atomic lattice near the Ni-HOPG interface. A relative compression of the nanoparticle atomic lattice *ξ* = 0.4–0.8% was shown to be enough for such an effect to manifest. Such compression increases the activation energy for oxygen incorporation by 6–12 kJ/mol, resulting in inhibition of oxide growth at the periphery of the nanoparticle. In fact, in this work, oxygen adatoms served as probes, and their incorporation between nickel atoms allowed the measurement of the nanoparticle’s lattice parameters at different distances from the Ni–HOPG interface. The developed theoretical framework not only accounts for the observed oxidation behavior but also offers a potential pathway to estimate charge transfer and local work functions for deposited nickel catalysts.

## 1. Introduction

The investigation of catalytic processes on solid surfaces is a challenging task. Often being non-equilibrium, these processes can involve stages of surface reconstruction, adsorbate migration, and occur with the participation of electronically and vibrationally excited active sites [1]. Consequently, local reactions and product accumulation rates can vary significantly [2]. Sample preparation with a high degree of surface homogeneity only partially helps to solve this problem. Furthermore, such samples have little relevance to real catalytic systems [3]. One possible solution to this problem is the use of scanning tunneling microscopy and spectroscopy (STM/STS) techniques, which, despite some limitations, allow for the acquisition of detailed information about the distribution of reaction products on the surface of even a single nanoparticle [4]. 

Using these methods, we investigated the oxidation of nickel nanoparticles deposited on highly oriented pyrolytic graphite (HOPG) in O_2_ [5]. It was shown that the oxide forms only at the top of the nanoparticle. The area near the Ni–HOPG interface exhibits resistance to oxidation. Initially, we hypothesized that electron transfer from the support to the nanoparticle limits oxygen diffusion in this system. However, in that case, the oxidation-resistant area should correlate in size with the charge redistribution area, meaning it should be significantly smaller, considering the very small difference in work function between nickel and graphite. Subsequently, we found no further evidence for this hypothesis.

In our research we successfully linked the adsorption characteristics of gold, nickel, and platinum, comparing their behavior in bulk form to that of nanoparticles deposited on highly oriented pyrolytic graphite (HOPG) [6]. Using STM/STS techniques, experiments with limited gas exposure revealed a significant variation in adsorption behavior between the top of the nanoparticles and the area close to the metal–HOPG interface. These experiments identified factors influencing adsorption and, within the framework of the Newns-Anderson chemisorption model, qualitatively described the formation of a stable surface adsorption complex Me–Ads [6].

The difference in adsorption properties on the surface of a single nanoparticle is due to the varying local contributions of the nanoparticle’s charging, the deformation of its atomic lattice, and the hybridization of surface electronic states. These results allow for a qualitative assessment of the adsorption properties of nanostructured systems, taking into account the electron work function, plasticity, and electronic structure of bulk materials.

Some works in this series were dedicated to hydrogen adsorption on gold nanoparticles. The locality of the surface filling process with adsorbate was experimentally proved: hydrogen adsorption begins near the graphite–gold interface [7]. Furthermore, the threshold size of gold nanoparticles determining the change in adsorption properties was determined [8]. Hydrogen is dissociatively chemisorbed on nanoparticles 5–6 nm in diameter, while increasing the size to 10 nm or more leads to inhibition of chemisorption.

The studies were continued on platinum nanoparticles [6], which allowed for the evaluation of the influence of the hybridization of surface *s*- and *p*-states on the local stability of the Pt–O adsorption complex. Experiments showed that the adsorption properties of large platinum particles are similar to those of nickel particles: the Pt–O adsorption complex is more stable away from the Me–HOPG interface. This is predictable, as the *d*-band states contribute to the formation of the complex. However, for platinum nanoparticles, the opposite result is observed: the Pt–O complex is more stable near the platinum-graphite interface. Increasing the contribution of the hybrid *sp*-band makes platinum nanoparticles more similar to gold nanoparticles in their adsorption properties.

We can conclude that the comparative study [6] effectively describes the qualitative trends for all three investigated systems, but many questions remain unanswered.

In the Ni–HOPG system, the contribution of the work function difference to the surface activity will be much smaller than in the Au–HOPG and Pt–HOPG ones [9]. Therefore, we can take the next step—to hypothesize that the change in surface activity of nickel nanoparticles is determined to a greater extent by a structural factor, and to experimentally estimate the degree of lattice deformation of the nanoparticle. We will attempt to address this task in this article.

## 2. Experimental

The experiments were performed in the chamber of an ultra-high vacuum (UHV) scanning tunneling microscope (VT STM, Omicron NanoTechnology, Taunusstein, Germany). The residual pressure in the UHV chamber did not exceed 2⋅10^−10^ Torr, which allowed us to avoid uncontrolled chemical changes to the sample surfaces due to interaction with residual gases. The sample exposure was measured in Langmuirs (1 L = 10^−6^ Torr⋅s). N_2_O was used as the oxidizing agent, and its injection into the system was carried out using a special gas dosing system. The composition of the gas atmosphere inside the setup was monitored using a quadrupole mass spectrometer (HAL 301 PIC, Hiden Analytical Limited, Warrington, UK).

In the STM/STS experiments, we used tips fabricated by cutting a taut wire made of a platinum-iridium alloy. Tips were deemed suitable for experiments if, when scanning the HOPG surface, they exhibited a reproducible S-shaped current-voltage curve (CVC) for the tunneling junction. This type of CVC is characteristic of metal–metal tunnel junctions [10].

The synthesis of nanoparticles was performed in situ within the scanning tunneling microscope chamber using a standard impregnation method. An aqueous solution of nickel nitrate, Ni(NO_3_)_2_ (Moscow State University, Moscow, Russia), with a metal concentration of 24 mg/L was used as the precursor. The precursor solution was deposited onto a cleaned HOPG surface (HOPG, AIST-NT, Moscow, Russia), which exhibited extensive atomically smooth C(0001) terraces [11]. After drying the solution, the sample was placed into the STM chamber, where it was annealed in hydrogen (*p* = 10^−6^ Torr) at *T* = 950 K for 40 h.

The decomposition of the precursor proceeds as follows [12]:

(1) $$Ni{\left({NO}_{3}\right)}_{2}\overset{\sim 573\;K}{\to }NiO+O_{2}+{NO}_{2}$$

The subsequent reduction of metallic nickel by hydrogen can be described as follows:

(2) $$NiO+H_{2}\overset{\sim 535\;K}{\to }Ni+H_{2}O\;.$$

Thus, the chosen annealing temperature of the sample ensures the complete reduction of nickel. During annealing, all gaseous synthesis products were pumped out of the UHV chamber. After synthesis, the residual gas pressure in the UHV chamber corresponded to the standard level.

The synthesized nanoparticles were exposed to N_2_O (50 L) at *T* = 300 K under a pressure of *p* = 10^−6^ Torr in the UHV STM chamber. The exposure time was 50 s. Before the gas was introduced, the STM tip was retracted as far as possible from the sample surface. Approximately 5–6 h after the exposure and pumping out of the N_2_O from the UHV chamber, the structure of the oxidized nanoparticles was investigated using standard STM/STS methods. The condition of the tip was checked on clean HOPG areas before each measurement.

## 3. Results

**Figure 1:**
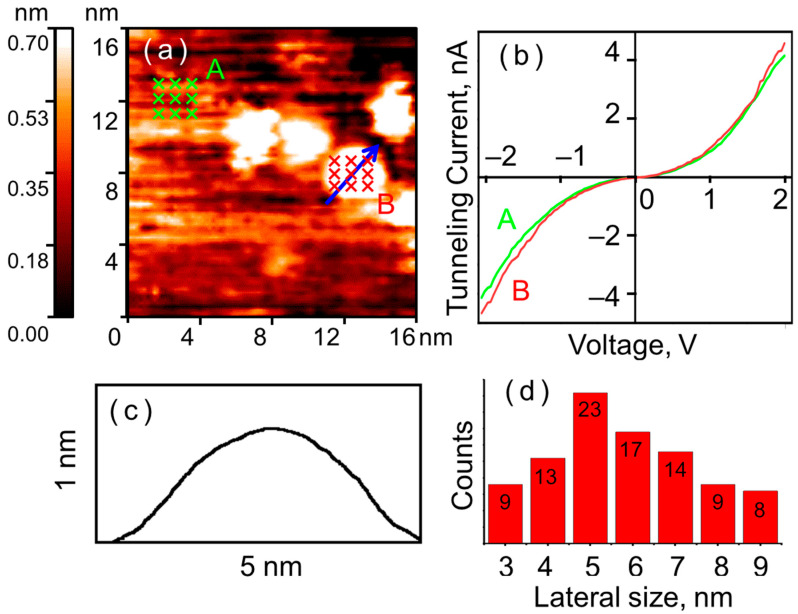
Nickel nanoparticles after annealing in UHV with simultaneous reduction in H_2_. STM measurement results: (**a**) topography of the HOPG surface with a single nickel nanoparticles; (**b**) CVCs of the tunnel junction on the HOPG surface (green curve A) and on the nanoparticle (red curve B), averaged over a set of points marked with crosses in (**a**); (**c**) surface profile along the cut line shown in (**a**) with a blue arrow; (**d**) lateral size distribution of investigated particles.

STM studies revealed that after annealing in H_2_, nanoparticles with a shape close to a flattened hemisphere are formed on the HOPG surface. The nanoparticles form agglomerates and decorate the edges of the HOPG terraces. The average lateral size of the particles is 5–6 nm (see Figure 1). The experimentally studied sample consisted of 93 particles, of which 40 had the specified size. Their average height relative to the atomically smooth HOPG terrace is 0.8–3.0 nm. Spectroscopic results indicate that the nanoparticle surface after annealing possesses a metallic electronic structure. All CVCs, measured at various points on the nanoparticles surface and on clean HOPG, exhibit a smooth, S-shaped form typical of metal–metal tunnel junctions. CVCs of this form were observed for all investigated nanoparticles.

**Figure 2:**
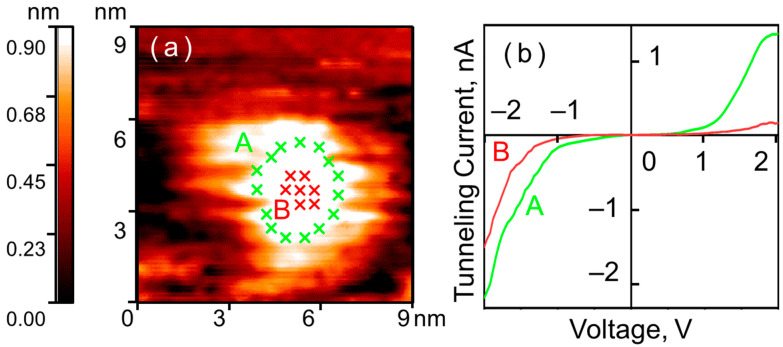
Nickel nanoparticle on HOPG after exposure to N_2_O (50 L, 10^−6^ Torr, 50 s): (**a**) surface topography; (**b**) averaged CVCs corresponding to the points indicated in the topography. Green curve A—nanoparticle surface near the Ni-HOPG interface; red curve B—central region of the nanoparticle.

Experiments on the oxidation of the nanoparticle surface in N_2_O were performed at a pressure of *p* = 10^−6^ Torr. The exposure was 50 L. The duration of the sample’s exposure to N_2_O was 50 s. After the exposure, the residual gas was pumped out of the UHV chamber. The oxidation results were experimentally studied on a sample of approximately 20 particles with a size of 5–6 nm. The STM measurements showed that the electronic structure of the nanoparticles changed significantly, and along with it, the shape of the CVCs. Two distinct types of electronic structure can be observed on the nanoparticle surface (see Figure 2).

On the CVCs measured in the central part of the nanoparticle—at its top—a zero-current region appears. Its extent is 1.1–1.8 V. This change in CVC shape during oxidation indicates the formation of a semiconductor oxide layer on the nanoparticle surface. Up to a dimensional factor, the extent of the zero-current region corresponds to the local band gap of the forming surface oxide [10]. Meanwhile, the nanoparticle surface along the visible edge retains its metallic conductivity type—its electronic structure does not change. The CVCs of this form and their distribution across the surface were observed for all investigated nanoparticles.

The observed change in the CVC shape allows us to draw the following conclusion. Exposure to 50 L of N_2_O at a pressure of *p* = 10^−6^ Torr leads to oxide formation only at the top of the nanoparticle. At the periphery of the nanoparticle—along the visible edge—nickel exhibits resistance to oxidation. This result is fully analogous to that observed during the exposure of nickel nanoparticles to O_2_ [5,6].

## 4. Discussion

### 4.1. Kinetics of N_2_O Decomposition

The issue of adsorbate presence on the nanoparticle surface requires separate consideration. Since the amount of nickel in our experiments does not exceed 50 ng, we cannot determine the amount of N_2_ desorbed from the nanoparticle surface or detect the Auger-electrons signal of nickel oxide and directly investigate the kinetics of N_2_O decomposition. In some cases, using STS methods, the presence of molecular adsorbates on the surface can be established—through characteristic periodic features in the CVCs [4]. In this particular case, we do not observe such features, but this by no means proves that molecular adsorbates are absent. Chemisorbed oxygen can be visualized by STM, but only on atomically smooth surfaces when scanning in constant-height mode [13]. The presence of chemisorbed oxygen on the surface can also be established from STS measurements, but this requires assumptions about the energy of the electronic state formed during chemisorption and the calculation of the tunneling transition amplitude using quantum mechanical methods.

To account for the contribution of various forms of adsorption, it would be more reasonable in this experiment to study the kinetics of N_2_O dissociation on the nickel single crystal surface. Of course, the application of certain models to nano-objects always needs to be verified separately. In the absence of other options, we will rely on the internal consistency of our approximations and on agreement with the results obtained for similar systems.

**Figure 3:**
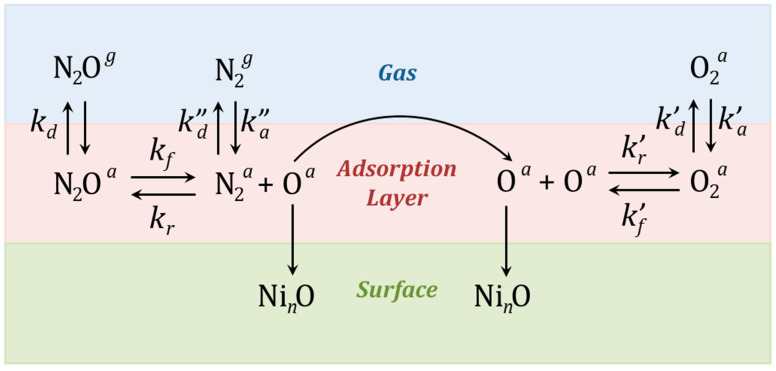
Interaction of N_2_O with the nickel nanoparticle surface. Processes of molecular adsorption and desorption, adatom formation, adatom recombination, surface migration, and incorporation into the metal lattice are shown. The scheme indicates the rate constants for those processes that can be described in terms of formal kinetics. Other processes will be discussed separately below.

Let us consider the interaction of N_2_O with the surface of a nickel single crystal (see Figure 3). Here, $k_{a}^{\prime }$ and $k_{a}^{\prime \prime }$ denote the adsorption rate constants,$\;k_{d}$, $k_{d}^{\prime }$ and $k_{d}^{\prime \prime }$ are the desorption rate constants, $k_{f}$ and $k_{f}^{\prime }$ are the fragmentation rate constants, and $k_{r}$ and $k_{r}^{\prime }$ are the recombination rate constants.

Molecular forms of oxygen adsorption are known only for a few systems and only at temperatures around 70 K [14]. Therefore, we can state that $k_{r}^{\prime }$ is negligibly small and, consequently, also exclude oxygen desorption from the surface from consideration. Conversely, $k_{d}^{\prime \prime }$ is very large [15,16]. The strong binding of oxygen to the surface indicates that $\;k_{r}$ is also very small, and the recombination of decomposition products is practically impossible [15,16].

It must be taken into account that the UHV chamber is a flow reactor in which the flux of gas molecules to the surface, *j*, can be considered constant; however, adsorption will strongly depend on the surface coverage of oxygen adatoms, *θ* [15,16].

The dependence of the sticking coefficient, *s*(*θ*), for N_2_O on single-crystal nickel can be approximately described by the following expression:

(3) $$s\left(\theta \right)=1-\;{\left(\frac{\theta }{\;\theta_{0}}\right)}^{2},$$

where the saturation limit is reached at $\theta =\theta_{0}=0.25$ [15,16]. We will discuss the accuracy of this approximation at the end of the section. The experimentally observed sticking coefficient deviates significantly from the linear dependence of 1 − *θ*/*θ*_0_, and practically does not change at *θ* < 0.125 ML [15,16]. This indicates the existence of a precursor—a pre-adsorbed state of the N_2_O molecule, in which it can move freely across the entire surface, including over adsorption sites already filled with oxygen [1]. This notably distinguishes the N_2_O molecule from O_2_, for which the dependence at *θ* < 0.25 ML is close to linear [17]. In works [15,16], the adsorption of the molecule on free and oxygen-filled sites was considered, using a model where dissociation occurs only on free sites. This model was successfully verified in the temperature range of 573–873 K, with the desorption energy for filled and free centers being 20.0 kJ/mol and 26.2 kJ/mol, respectively. However, it is worth noting that the authors failed to verify their model for temperatures of 323–432 K, even though the *s*(*θ*) dependencies were experimentally obtained. Perhaps the reason for this is that the model does not account for the surface migration of oxygen adatoms and, consequently, the exchange between adsorption sites of different types. More rigorous verification of the model requires studies that go beyond the scope of our work. The assumption that a free adsorption site is simultaneously a dissociation site seems quite reasonable to us. Since we are primarily interested in the molecule’s decomposition event, we can use this model, assuming that all information about the molecule’s ‘life’ prior to dissociation is already contained within the experimental *s*(*θ*) dependence. It can also be said that the observed similarity between the results of nickel oxidation in O_2_ and N_2_O is provided rather by the nature and evolution of the Me–Oads complex than by the presence of a molecular adsorbate. So we can describe kinetics of N_2_O decomposition by the following scheme:

(4) N2O g → j·sθ ← kd  N2O a → kf  O a + N2 a

The ratio *k_f_*/*k_d_* = 32.3 has been previously reported at *T* = 300 K [15]. Simultaneously, the activation energy *E_f_* = 10.7 kJ/mol, and the pre-exponential factor is *A*_0_ = 10^13^ s^−1^, which corresponds to monomolecular decomposition reactions [16]. By introducing the concentrations of adsorbed N_2_O molecules and oxygen adatoms on the surface, *C_M_* and *C_A_*, we can describe the processes on the surface with a system of differential equations:

(5) $$\left\{\begin{array}{c} \dot{{\;C}_{M}}=j\cdot s\left(\theta \right)-\left(k_{f}+k_{d}\right)\cdot C_{M}\;,\\ \dot{C_{A}}=k_{f}\cdot C_{M}\;. \end{array}\right.$$

At a pressure of 10^−6^ Torr and *T* = 300 K, the molecular flux can be estimated as *j* = 2⋅10^15^ molecules/cm^2^⋅s, and *k_f_* = 5 × 10^10^ s^−1^. The order of magnitude of the terms in the equations indicates that *C_M_* is very small and *Ċ_M_* ≈ 0. By introducing the adatom concentration in a monolayer, *C*_0_, and rewriting *s*(*θ*) as:

(6) $$s\left(C_{A}\right)=1-\frac{C_{A}^{2}}{\theta_{0}^{2}\cdot C_{0}^{2}}\;,$$

we can write the following:

(7) $$\dot{C_{A}}\;=\;\frac{k_{f}}{k_{f}+k_{d}}\cdot j\cdot {s(C}_{A})\;,$$

and thus we have:

(8) $$\int_{0}^{C_{A}}\frac{dC_{A}}{s(C_{A})}=jt\frac{k_{f}}{k_{f}+k_{d}}\;,$$

then we can write the following:

(9) $$\int_{0}^{C_{A}}\frac{dC_{A}}{s(C_{A})}=-\frac{\theta_{0}C_{0}}{2}\cdot \;ln\left|\frac{C_{A}-\theta_{0}C_{0}}{C_{A}+\theta_{0}C_{0}}\right|.$$

Finally, we obtain the result:

(10) $$C_{A}\left(t\right)=\theta_{0}C_{0}\cdot th\left[\frac{jt}{\theta_{0}C_{0}}\cdot \frac{k_{f}}{k_{f}+k_{d}}\right]\;.$$

By definition, *j* = *j*_0_·*p*/*p*_0_, where *j*_0_ is molecular flux at *p*_0_ = 10^−6^ Torr. It is more convenient to measure the adatom concentration in monolayers. Substituting the values *θ*_0_ = 0.25, *j*_0_ = 1 ML/s, *C*_0_ = 1 ML, and taking into account [15]:

(11) $$\frac{k_{f}}{k_{f}+k_{d}}\approx 1\;,$$

we obtain in numbers:

(12) $$C_{A}^{ML}\left(t\right)=0.25\cdot th\left(4\frac{p}{p_{0}}t\right)\;.$$

Surface saturation occurs very quickly. At N_2_O pressure of *p* = 10^−6^ Torr, within a time of *t* = 0.5 s, a coverage of *C_A_* = 0.241 ML forms on the surface. The amount of molecular N_2_O on the surface is negligible, as expected, *C_M_* = 1.4 × 10^−12^ ML. It should be noted that the experimentally observed sticking coefficient is more accurately described by a fourth-power dependence *s*(*θ*) = 1 − (*θ*/*θ*_0_)^4^. In this case, analytically obtaining the concentration-time dependence becomes somewhat more complicated. It can be shown numerically that at very low coverages, the slope of the $C_{A}^{ML}\left(t\right)$ curve at *t* = 0 does not change, and the aforementioned oxygen adatom concentration *C_A_* = 0.241 ML is reached in *t* = 0.35 s. This means that the process reaches its quasi-steady-state regime faster due to the existence of molecular adsorbates and their surface migration. It can be concluded that oxygen adatoms are present on the nickel nanoparticle surface, with a concentration of ≈0.25 ML. The decomposition of N_2_O on the surface reaches a stationary state (in terms of the molecular adsorbate concentration) in a time much shorter than the duration of the sample’s exposure to N_2_O.

This result is in good agreement with what we observed during the oxidation of nickel by oxygen. As we mentioned above, in the case of oxygen, the influence of molecular adsorbates is absent at *θ* < 0.25. It is known that during nickel oxidation in O_2_, the concentration of oxygen adatoms on the surface reaches 0.25 ML at a pressure of *p* = 10^−6^ Torr with an exposure of ∼20 L [17]. Since N_2_O dissociation yields twice less oxygen adatoms, it can be concluded that the obtained estimates are in good agreement with the literature. Furthermore, identical central-peripheral oxide distributions on the nanoparticle surface are observed during oxidation in O_2_ and N_2_O. In other words, the N_2_O adsorption on oxygen-filled sites accelerates surface saturation and does not qualitatively change the decomposition kinetics.

### 4.2. Oxidation Mechanism

The saturation of the metal surface with oxygen adatoms, by itself, does not lead to oxide formation. This is a more complex process that involves surface migration of oxygen adatoms, island growth of the surface oxide, and an increase in the oxide layer thickness [17]. For different metals, this last stage can occur via various mechanisms. For example, the increase in oxide thickness on the surface of iron occurs via an interchange mechanism (the Lanyon-Trapnell mechanism) [18,19]. In this case, an oxygen atom exchanges places with an underlying metal atom. This mechanism is characteristic of metals with a body-centered cubic lattice (Cr, Fe, Mo, W).

The oxidation of cobalt, nickel, and other metals with a face-centered cubic lattice occurs differently—through a stage of oxygen incorporation between metal atoms (the Burshstein-Shurmovskaya mechanism) [18,19]. In this case, the size of the interatomic gap into which the oxygen adatom penetrates is critical. If the gap is too small, oxygen incorporation can only occur in the case of vibrational excitation of the neighboring metal atoms. It is through this mechanism that nickel oxidation occurs [20,21]. For example, on the Ni(001) surface, the incorporation site is the gap between four nickel atoms, and this group of atoms must be vibrationally excited.

In the simplest approximation, an island of surface oxide can be represented as an area of the surface where the concentration of oxygen adatoms slightly exceeds the saturation concentration. Then, oxygen incorporation at the initial stages will be determined by the characteristic time of the adatom residence at the incorporation site and the characteristic time of the site relaxation. Let us consider such a problem.

### 4.3. Surface Migration of Adatom

Let an adatom be located within a potential well of the periodic surface potential at the initial moment. Interaction with phonons leads to the equilibrium energy distribution within this well, so the particle has a finite probability of transition into a higher energy state in which it can make a jump—move along the surface while remaining bound to it [1]. Let *τ_a_* be the characteristic time of the adatom residence at the incorporation site. Then, the characteristic time of the adatom jump will be:

(13) $$\tau_{a}^{*}=\tau_{a}\cdot exp\left(-\frac{E_{M}}{k_{B}T}\right)\;,$$

where *E_M_* is the activation energy for surface migration, and *k_B_* is the Boltzmann constant. In the same way, we can describe the characteristic time of excitation *τ_C_* and relaxation $\tau_{C}^{*}$ of an active site on the surface:

(14) $$\tau_{c}^{*}=\tau_{c}\cdot exp\left(-\frac{E_{C}}{k_{B}T}\right)\;,$$

where *E_C_* is the excitation energy. In this case, the characteristic excitation time of the site, *τ_C_*, represents the lifetime in the ground state, and the relaxation time, $\tau_{C}^{*}$, represents the lifetime of the excited state. Thus we can describe the excitation and relaxation of the site using a simple two-level approximation. The time required for the active site to incorporate an adatom can be estimated as follows:

(15) $$\tau_{i}≃\frac{\Delta h}{v}=\frac{\Delta h}{\lambda }\cdot \tau_{a}^{*}\;,$$

where Δ*h* is the penetration depth of the adatom into the active site, and *v* is the average velocity of the adatom on the surface.

For adatom penetration, the time of the adatom residence at the site must be sufficiently long for the site to become excited and incorporate the adatom:

(16) $$\tau_{a}>\tau_{c}+\tau_{i}\;,$$

which allows us to write the condition for oxide formation explicitly:

(17) $$\tau_{a}^{*}\left[exp\left(\frac{E_{M}}{k_{B}T}\right)-\frac{\Delta h}{\lambda }\right]>\tau_{c}^{*}\cdot exp\left(\frac{E_{C}}{k_{B}T}\right)\;.$$

Clearly, the ratio Δ*h*/λ will be negligibly small compared to the exponential terms. This allows us to simplify the final expression:

(18) $$\tau_{a}^{*}\cdot exp\left(\frac{E_{M}}{k_{B}T}\right)>\tau_{c}^{*}\cdot exp\left(\frac{E_{C}}{k_{B}T}\right)\;.$$

The characteristic times $\tau_{a}^{*}$ and $\tau_{c}^{*}$ can differ significantly, so it is incorrect to simply compare the energies *E_M_* and *E_C_*.

### 4.4. Activation Energy of Adatom Incorporation

Data on the surface migration of heteroatoms for various nickel surfaces are scarce [22]. A DFT calculation for the (111) facet yields a migration activation energy of *E_M_* = 57 kJ/mol for oxygen adatoms [23]. The results of work [24] are considered reliable—the only known experimental study where a migration activation energy of *E_M_* = 55 kJ/mol was determined for a flat nickel (001) surface. In this work, a short-jump model was initially used to estimate the activation energy, and the authors themselves note that they could only observe slow processes. But, overall, all the obtained values are close to the standard value for such processes, *E_M_* ≈ 60 kJ/mol [1].

The situation is much more complicated with the incorporation activation energy, *E_C_*. First, it will depend on the size of the interatomic gap into which the oxygen is incorporated, and therefore will depend on the strain of the nanoparticle’s crystal lattice. Second, it will depend on the surface coverage with oxygen adatoms, *θ*. Since each adatom carries some charge, it will change the potential in which the nickel atoms move on the surface. At low coverage of adatoms at *T* = 273 K and without strain, *E_C_* = 30 kJ/mol for the nickel (001) surface [20]. The correction due to surface coverage, *ε*(*θ*), can be described by a linear dependence:

(19) $$\varepsilon \left(\theta \right)=-\varepsilon_{0}\cdot \theta \;,$$

where *ε*_0_ = 23 kJ/mol [20]. To describe the dependence of *E_C_* on strain, we take advantage of the fact that the strain is small.

### 4.5. Nickel Lattice Deformation

**Figure 4:**
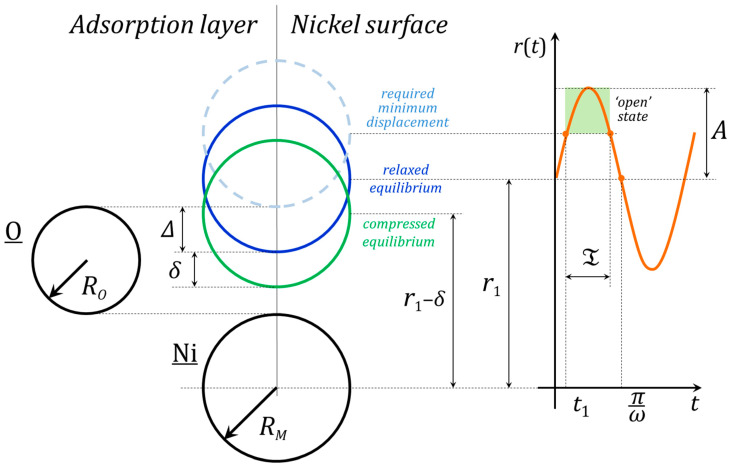
Diagram of nickel atoms motion in approximation of a harmonic oscillator with vibration amplitude *A* and frequency *ω*. Interatomic distances for active site in the case of a relaxed and compressed nickel lattice and required minimum displacement are shown. An oxygen adatom can incorporate between nickel atoms if the upper atom is displaced from its equilibrium position (blue circle) by Δ (dashed light blue circle). This is possible if the energy $E_{C}$ is concentrated at the active site, and nickel atom position is *r*(*t*) > *r*_1_ + Δ for the time T. If the lattice is compressed, the upper nickel atom must overcome the resulting deformation, i.e., it must displace by Δ + δ from its new equilibrium position (green circle).

At small strains, we can describe the motion of the atoms of the oxygen incorporation site in harmonic oscillator approximation (see Figure 4). Let the characteristic interatomic distance for an incorporation site without strain be *r*_1_. Then, the position of nickel atom will be:

(20) $$r\left(t\right)=r_{1}+A\cdot \sin\omega t\;,$$

where *A* and *ω* are vibrational amplitude and frequency correspondingly. Taking into account the zero-point vibration, one can write:

(21) $$E_{C}+\frac{\hbar \omega }{2}=\frac{A^{2}M\omega^{2}}{2},$$

wherwhere *M* is the reduced mass, and *E_C_* is the activation energy of oxygen incorporation. Incorporation takes place when the oscillator is ‘open’, which is described by following condition:

(22) A·sinωt≥∆ ,

where Δ < *A* is the required minimal displacement of the nickel atom. If the ‘opening’ of the site starts at moment *t*_1_, then in first approximation it is:

(23) $$t_{1}≅\frac{∆}{A\omega }\;,$$

and the ‘open’ period duration is:

(24) $$T=\frac{\pi }{\omega }-\frac{2}{\omega }\cdot \frac{∆}{A}\;.$$

The same is valid for compressed lattice:

(25) $$r^{\prime }\left(t\right)=r_{1}-\delta +\left(A+\delta \right)\cdot \sin\omega t\;,$$

(26) $$E_{C}^{\prime }+\frac{\hbar \omega }{2}=\frac{{\left(A+\delta \right)}^{2}M\omega^{2}}{2}\;,$$

(27) $$T^{\prime }=\frac{\pi }{\omega }-\frac{2}{\omega }\cdot \frac{∆+\delta }{A+\delta }\;,$$

where *δ* > 0 is a decrease in characteristic interatomic distance. Then one can write:

(28) $$\frac{E_{C}^{\prime }-E_{C}}{E_{C}}={\left(\frac{A+\delta }{A}\right)}^{2}-1.$$

If compressed lattice works at $E_{C}^{\prime }$ as efficiently as relaxed lattice at $E_{C}$, for ‘open’ state duration we can consider $T≅T^{\prime }\;,$ and so we have:

(29) $$\frac{∆+\delta }{A+\delta }≅\frac{∆}{A}\;.$$

Introducing the parameter α=δ/Δ≪1, let us rewrite Equation (28):

(30) $$\frac{E_{C}^{\prime }}{E_{C}}=1+2\alpha +o\left(\alpha \right)\;.$$

Thus, we have established a relationship between the oxygen incorporation energy and some parameter of the site deformation, α, which remains unknown to us for now.

Let us try to estimate how the number of excited active sites on the nanoparticle surface will change upon compression of its crystal lattice. Let the total number of active incorporation sites be *Z*_0_. Thus the excited ones among them will be:

(31) $$Z_{C}=\left(Z_{0}-Z_{C}\right)\cdot exp\left[-\frac{E_{C}+\varepsilon \left(\theta \right)}{k_{B}T}\right]≅Z_{0}\cdot exp\left[-\frac{E_{C}+\varepsilon \left(\theta \right)}{k_{B}T}\right]\;,$$

where *ε*(*θ*) is an energy correction that depends on the surface coverage with oxygen adatoms, *θ*. Here, we have already utilized the fact that *Z_C_* ≪ *Z*_0_. We can write a similar expression for a nanoparticle with a compressed lattice:

(32) $$Z_{C}^{\prime }≃Z_{0}\cdot exp\left[-\frac{E_{C}^{\prime }+\varepsilon \left(\theta \right)}{k_{B}T}\right]\;.$$

The total number of active sites is determined solely by the atomic packing in the lattice and does not depend on the strain. Therefore, we write *Z*_0_ on the right-hand side. Then we can say:

(33) $$\frac{Z_{C}^{\prime }}{Z_{C}}=exp\left[-\frac{2\alpha E_{C}}{k_{B}T}\right]\;.$$

One can see that the relative error in determining α will be much smaller than the relative error of the expression on the left-hand side. In this case, even a rough estimate of the ratio *Z*′_C_/*Z*_C_ will be sufficient.

The total number of active sites on the nanoparticle surface will be approximately equal to the number of surface atoms. For a hemisphere nickel nanoparticle with a diameter of ~5 nm, it amounts to *Z*_0_ ≅ 10^3^. Lattice compression reduces the number of excited sites, but oxidation would be impossible even with long exposures, if they were to disappear completely. Given that *Z_C_*, *Z*′*_C_* ≪ *Z*_0_, we can roughly estimate:

(34) $$\frac{Z_{C}^{\prime }}{Z_{C}}≃10^{-2}-10^{-1}\;.$$

Then, substituting the value *E_C_* = 30 kJ/mol at *T* = 300 K, we obtain:

(35) $$\alpha =-\frac{k_{B}T}{2E_{C}}\cdot ln\left(\frac{Z_{C}^{\prime }}{Z_{C}}\right)=0.1-0.2\;.$$

The required value of Δ is determined by the geometry of the incorporation site, the atomic radius of oxygen *R_O_* = 0.68 Å, and the covalent radius of nickel *R_M_* = 1.15 Å [9]. The lattice parameter is *a* = 3.524 Å for face-centered cubic nickel lattice [9]. Then:

(36) $$\Delta =2R_{O}-\left(a-2R_{M}\right)=0.14\;Å\;.$$

Thus we obtain:

(37) δ=α·∆=0.014−0.028 Å .

Since the interatomic distance at the incorporation site is $r_{1}=a=3.524\;Å$, one can calculate the relative lattice compression:

(38) $$\xi =\delta /r_{1}=0.4-0.8\;\%.$$

Let us compare the obtained result with data found in the literature. There are known studies that investigated nickel nanoparticles placed inside nanotubes using the TEM method [25,26]. Knowing the lattice constant *a_c_* = 1.421 Å for C(0001), the theoretically expected compression of the (111) facet in such a case is *ξ* = 1.21%—this value is close to our maximum estimate. The experimentally observed value for the Ni(111) is *ξ* = 0.35%—this value is close to our minimum estimate. It should be recalled that in our rather rough estimations, the HOPG lattice parameters do not appear at all—we only considered the probability of oxygen adatom incorporation into the nickel lattice. Considering the above, our obtained value matches the literature data surprisingly well. In our model, the obtained relative change in the number of excited sites *Z*′_C_/*Z*_C_ ≅ 10^−2^–10^−1^ and a value of α = 0.1–0.2 result in an increase of 6–12 kJ/mol in the activation energy for oxygen incorporation. 

### 4.6. Active Site Relaxation

Now that we have made all the necessary estimations, we can return to the previously obtained condition for oxide formation (18) and check how well it is satisfied. At the top of the nanoparticle, we will consider the strain contribution to be negligible, and therefore only account for the oxygen coverage correction. We mentioned that during surface migration, an area with an adatom concentration *θ*′ > *θ*_0_ can form, where oxide growth begins. For nickel single crystals, it is known that uniform growth of the oxide film begins at *θ*′ = 0.35 [17]. Then, for the top of the nanoparticle, we can write:

(39) $$\tau_{a}^{*}\cdot exp\left(\frac{E_{M}}{k_{B}T}\right)>\tau_{c}^{*}\cdot exp\left(\frac{E_{C}-\varepsilon_{0}\cdot \theta^{\prime }}{k_{B}T}\right)\;.$$

At the periphery of the nanoparticle, the oxide does not form. The local adatom concentration can reach a value of *θ*′, but here it is necessary to consider the strain contribution due to interaction with HOPG. One can write:

(40) $$\tau_{c}^{*}\cdot exp\left(\frac{E_{C}^{\prime }-\varepsilon_{0}\cdot \theta^{\prime }}{k_{B}T}\right)\;>\;\tau_{a}^{*}\cdot exp\left(\frac{E_{M}}{k_{B}T}\right)\;.$$

Let us substitute $E_{C}^{\prime }=E_{C}\left(1+2\alpha \right)$ and bring everything together:

(41) $$exp\left(\frac{E_{C}\left(1+2\alpha \right)-\varepsilon_{0}\cdot \theta^{\prime }-E_{M}}{k_{B}T}\right)>\;\frac{\tau_{a}^{*}}{\tau_{c}^{*}}>exp\left(\frac{E_{C}-\varepsilon_{0}\cdot \theta^{\prime }-E_{M}}{k_{B}T}\right)\;.$$

For *α* = 0.1–0.2 and *E_M_* = 55 kJ/mol we obtain numerically:

(42) $$2\cdot 10^{-4}>\frac{\tau_{a}^{*}}{\tau_{c}^{*}}>2\cdot 10^{-6}\;.$$

We can estimate the time $\tau_{a}^{*}$ by knowing λ = 10^−8^ cm and *v* = 10^4^–10^5^ cm/s [1]. We obtain:

(43) $$\tau_{a}^{*}=\lambda /v=10^{-13}-10^{-12}s\;,$$

which corresponds to standard time for monomolecular processes. Then, for the time $\tau_{c}^{*}$ it is obvious that:

(44) $$2\cdot 10^{-9}s<\tau_{c}^{*}<2\cdot 10^{-7}s\;.$$

The obtained value of $\tau_{c}^{*}$ fits well within the range of characteristic relaxation times of vibrationally excited molecules on the surface of a solid [1]. This result once again confirms the adequacy of the models used in the work.

## 5. Conclusions

In this work, we have continued our studies of the local chemical activity of the surface of nickel nanoparticles, initiated in previous publications. Using STM/STS methods, we were able to observe different oxidation resistance in N_2_O at the top and periphery of nickel nanoparticles deposited on HOPG. 

It was shown that at an N_2_O pressure of *p* = 10^−6^ Torr, an exposure of 50 L leads to the formation of an oxide only at the apex of the nanoparticle. At the periphery, near the Ni-HOPG interface, the surface of the nanoparticle exhibits resistance to oxidation.

A significant result of our study was a detailed examination of the mechanism of the nanoparticle surface oxidation. The rate-limiting step in this case is the incorporation of oxygen into the nickel lattice. The active site for oxygen incorporation is a vibrationally excited group of several nickel atoms, the gap between which is penetrated by an oxygen adatom. In fact, in this work, oxygen adatoms served as probes, and their incorporation between nickel atoms allowed the measurement of the nanoparticle’s lattice parameters at different distances from the Ni–HOPG interface. Within the framework of a rather simple model, we have described the dependence of the incorporation activation energy *E_C_* on the relative lattice strain *ξ* and the surface coverage by oxygen adatoms *θ*. Near the Ni-HOPG interface, the relative compression of the nanoparticle’s lattice was estimated to be *ξ* = 0.4–0.8%, which is close to known experimental data. The activation energy for oxygen incorporation increases by 6–12 kJ/mol under such compression, which inhibits the growth of the oxide at the nanoparticle periphery. Also, numerical estimates were made for the characteristic time of vibrational relaxation of the active site, which is $\tau_{c}^{*}\;=10^{-9}\;-\;10^{-7}s$. The obtained value is in full agreement with the literature data.

We consider the obtained result to be quite interesting. Due to the very small work function difference between Ni and HOPG, we have observed a rather subtle structural effect. Nickel is unique in this respect. For other metals with face-centered cubic lattice (Pt, Pd, Ag, Rh, Ir), the calculated Δ is 3–4 times smaller, meaning the necessary atomic displacement is covered by the zero-point vibration amplitude. In the case of Al, the calculated Δ is negative, and it experiences no issues with oxygen incorporation. The calculated Δ for Cu is twice as large, implying a significantly higher activation energy, and oxidation proceeds via a different mechanism. This likely explains why we were unable to observe the central-peripheral oxide distribution for copper nanoparticles. The involvement of a low-probability event—the opening of a ‘window’ in the lattice—may explain the selectivity of processes that cannot be accounted for by charge effects.

It should be noted that condition (41) can also account for the charging effect from the substrate, similar to the correction for the oxygen adatom charge. Consequently, when nickel nanoparticles are deposited onto different substrates, these effects can either reinforce or compensate each other. Hypothetically, this scenario enables us to estimate the charge transferred to the nickel nanoparticle. For supports with significantly inhomogeneous surfaces, this also allows for the estimation of the local work function by watching the specific distribution of nickel oxide.

It is also noteworthy that we did not need to resort to any specific nano-assumptions to explain the effect. All models are size-independent. This further underscores that the applicability of models to a given object should be determined not by size, but by parameters that enable the establishment of a hierarchy for the occurring processes. In this context, the characteristic time of active site vibrational relaxation serves as such a parameter.

## Data Availability

The original contributions presented in this study are included in the article. Further inquiries can be directed to the corresponding authors.
